# Systematic Review of Single-Fraction Stereotactic Body Radiation Therapy for Early Stage Non-Small-Cell Lung Cancer and Lung Oligometastases: How to Stop Worrying and Love One and Done

**DOI:** 10.3390/cancers14030790

**Published:** 2022-02-03

**Authors:** Austin J. Bartl, Mary Mahoney, Mark W. Hennon, Sai Yendamuri, Gregory M. M. Videtic, Kevin L. Stephans, Shankar Siva, Mark K. Farrugia, Sung Jun Ma, Anurag K. Singh

**Affiliations:** 1 Jacobs School of Medicine and Biomedical Sciences, University at Buffalo, The State University of New York, Buffalo, NY 14203, USA; abartl@buffalo.edu; 2 College of Medicine, State University of New York Upstate Medical University, Syracuse, NY 13210, USA; mahonmar@upstate.edu; 3 Department of Thoracic Surgery, Roswell Park Comprehensive Cancer Center, Buffalo, NY 14203, USA; mark.hennon@roswellpark.org (M.W.H.); sai.yendamuri@roswellpark.org (S.Y.); 4 Department of Radiation Oncology, Cleveland Clinic Taussig Cancer Institute, Cleveland, OH 44195, USA; videtig@ccf.org (G.M.M.V.); stephak@ccf.org (K.L.S.); 5 Department of Radiation Oncology, Peter MacCallum Cancer Centre, Melbourne, VIC 3000, Australia; shankar.siva@petermac.org; 6 Department of Radiation Medicine, Roswell Park Comprehensive Cancer Center, Buffalo, NY 14203, USA; mark.farrugia@roswellpark.org (M.K.F.); sungjun.ma@roswellpark.org (S.J.M.)

**Keywords:** lung, NSCLC, lung oligometastases, SABR, SBRT, single fraction

## Abstract

**Simple Summary:**

Adoption of single-fraction stereotactic body radiation therapy for lung tumors has been limited among different institutions even during the coronavirus disease 2019 (COVID-19) pandemic. Potential reasons may include a lack of familiarity and concerns regarding potential toxicities. To address this knowledge gap, we performed a systematic review of prospective literature on single-fraction SBRT. Our PubMed search of prospective trials resulted in nine studies that showed favorable local control and survival outcomes among peripheral tumors. Many toxicities were grade 1–2, with 0–17% of patients experiencing grade ≥3 toxicity. Encouraging outcomes suggest that the utility of single-fraction stereotactic body radiation therapy may extend beyond the current COVID-19 pandemic.

**Abstract:**

Adoption of single-fraction lung stereotactic body radiation therapy (SBRT) for patients with medically inoperable early stage non-small-cell lung cancer (NSCLC) or oligometastatic lung disease, even during the coronavirus disease 2019 (COVID-19) pandemic, was limited despite encouraging phase II trial results. Barriers to using single-fraction SBRT may include lack of familiarity with the regimen and lack of clarity about the expected toxicity. To address these concerns, we performed a systematic review of prospective literature on single-fraction SBRT for definitive treatment of early stage and oligometastatic lung cancer. A PubMed search of prospective studies in English on single-fraction lung SBRT was conducted. A systematic review was performed of the studies that reported clinical outcomes of single-fraction SBRT in the treatment of early stage non-small-cell lung cancer and lung oligometastases. The current prospective literature including nine trials supports the use of single-fraction SBRT in the definitive treatment of early stage peripheral NSCLC and lung oligometastases. Most studies cite local control rates of >90%, mild toxicity profiles, and favorable survival outcomes. Most toxicities reported were grade 1–2, with grade ≥3 toxicity in 0–17% of patients. Prospective trial results suggest potential consideration of utilizing single-fraction SBRT beyond the COVID-19 pandemic.

## 1. Introduction

During the coronavirus disease 2019 (COVID-19) pandemic, treatment recommendations for cancer have emphasized delivering high-quality care while minimizing in-person interactions between patients and healthcare providers [1]. For medically inoperable patients with early stage non-small cell lung cancer (NSCLC) or lung oligometastases, a range of treatment guidelines has supported stereotactic body radiation therapy (SBRT) [2,3,4,5,6]. Several prospective studies have substantiated the therapeutic utility of SBRT as a surgical alternative, with long-term local control rates of ~90% at 5 years [7,8,9]. Prospective trials [10,11] and retrospective studies [12,13,14] showed similar outcomes for NSCLC patients treated with single- and multi-fraction SBRT. The National Comprehensive Cancer Network (NCCN) guidelines support single-fraction lung SBRT for peripheral NSCLC [15]. The lungs are the first site of distant metastatic disease for many malignant tumors. A randomized trial in pulmonary oligometastases suggested similar outcomes at 1 year post single- or multi-fraction SBRT [16].

Despite this evidence, prior to and even during COVID-19, adoption of single-fraction SBRT was modest [17,18,19]. Potential explanations include: lack of familiarity with the evidence and lack of clarity about the expected toxicity [4]. To address these concerns, we performed a systematic review of prospective literature on single-fraction SBRT for definitive treatment of early-stage and oligometastatic lung cancer patients.

## 2. Materials and Methods

A comprehensive PubMed search of published journal articles written in English related to single-fraction lung SBRT was performed using keywords “single fraction”, “stereotactic body radiation therapy”, “SBRT”, “SABR”, “lung cancer”, “NSCLC”, and “oligometastatic”. The inclusion criteria were (1) prospective studies and (2) studies citing clinical outcomes on early stage NSCLC or lung oligometastases definitively treated with SBRT in a single fraction. Trials comparing several fractionation schedules that included a single-fraction regimen, and studies not available in the PubMed database but satisfying the inclusion criteria, were also included in this review. Exclusion criteria were (1) retrospective studies, case reports, meta-analyses, or review articles; (2) studies only using multi-fractionated SBRT regimens; and (3) studies involving carbon ion radiotherapy. Our report follows the Preferred Reporting Items for Systematic Reviews and Meta-Analyses (PRISMA) guideline [20]. The literature search was conducted and completed in October 2021. Three reviewers determined the eligibility of studies for inclusion based on the above selection criteria.

## 3. Results

**Figure 1:**
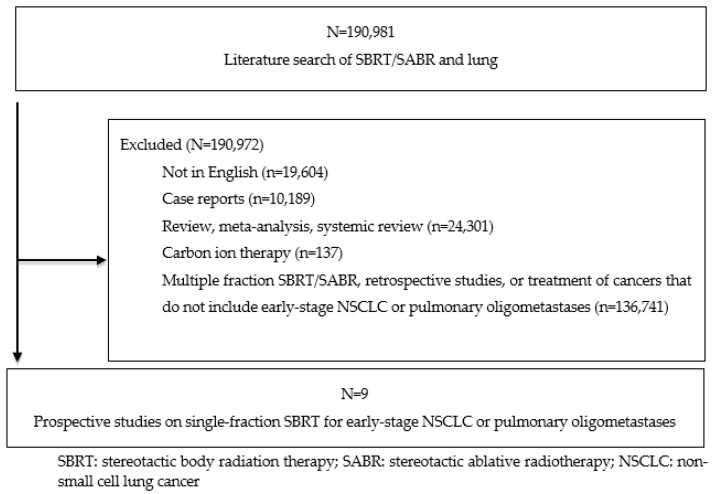
Study selection flow diagram.

Nine studies met criteria for this analysis [10,11,16,21,22,23,24,25,26]. Publication years ranged from 2003 to 2021. The PRISMA decision tree is shown in Figure 1.

### 3.1. Medically Inoperable Early Stage NSCLC: Local Control and Survival

Early single-fraction publications addressed the feasibility, safety, and efficacy of this SBRT schedule, with outcomes showing encouraging local control (LC) rates, tolerable toxicity profiles, and low rates of distant failures [22,23,25]. Mature studies of single-fraction SBRT for early stage NSCLC confirmed the earlier excellent rates of local control and minimal rates of grade 3 or higher toxicity [10,11]. Table 1 summarizes each study’s characteristics and outcomes.

Radiation Therapy Oncology Group (RTOG) 0915 randomized 84 medically inoperable patients with peripheral T1-2N0M0 NSCLC to receive either 34 Gray (Gy) in a single fraction or 48 Gy over four consecutively delivered fractions [11]. There were no significant differences in 5 year primary tumor failure rates (11%; 7%), distant failures (38; 41%), median progression-free survival (PFS; 2.6 and 2.8 years), and median overall survival (OS; 4.1 and 4.6 years) for 34 and 48 Gy, respectively [11].

Singh et al., in a multi-institutional phase II trial, randomized 98 medically inoperable patients with peripheral T1-2N0M0 NSCLC to 30 Gy in a single fraction or 60 Gy in three fractions [10]. There were no significant differences in 2 year rates of: LC (94.9% and 97.1%), regional nodal failure (8% and 16%), distant failure (13% and 19%), PFS (65% and 50%), or OS (73% and 62%) [10].

### 3.2. Lung Oligometastases: Local Control and Survival

In prospective trials investigating lung oligometastases, tumor control, regression, relapse, distant-site failure, DFS, PFS, and OS, results were collected and documented. Tumor control measures and survival outcomes are listed in Table 2.

Hof et al. treated 61 metastatic patients with single-fraction SBRT ranging 12–30 Gy [24]. Median follow-up time was 14 months [24]. Long-term local tumor control was not achieved for metastases treated with <24 Gy, whereas the local progression-free rate (LPFR) for metastases treated with ≥24 Gy was 78% after 36 months [24]. Additionally, there was a trend of better local control for tumors ≤10 cm^3^ at a follow-up of 3 years, and the difference became more evident with radiation ≥26 Gy [24].

Filippi et al. treated 67 patients with peripherally located metastatic lung tumors with 26 Gy in a single fraction [21]. Median follow-up time was 24 months [21]. Local failures were noted in 11% of patients [21]. Systemic failure occurred in 55% of patients at a median of 8 months after radiation, with no difference in the crude systemic failure rate between patients with a single tumor and those with 2–3 metastases [21].

The phase II SAFRON II trial randomized 87 patients with 133 pulmonary metastases to either 28 Gy in a single fraction or 48 Gy in four fractions [16]. No significant differences were found between the multi- and single-fraction arms for freedom from local failure (95% versus 93%, *p* = 0.13), OS (95% versus 93%, *p* = 0.44), or median disease-free survival (13.2 versus 14.3 months, *p* > 0.99) [16].

### 3.3. Toxicity of SBRT in Primary NSCLC and Lung Oligometastases

Table 3 summarizes the reported toxicity in these studies for both early stage NSCLC and lung oligometastases. It demonstrates overall favorable toxicity profiles with single-fraction SBRT.

The primary endpoint of RTOG 0915 was the rate of grade ≥3 protocol-specified adverse events (psAEs) at 1 year. Grade 3 or higher toxicities were 2.6% with 34 Gy and 11.1% with 48 Gy [11]. Based on pre-specified rules, since there was less nominal toxicity with comparable primary tumor control, single fraction was selected as the arm for future study [11]. 

Singh et al. used rates of Common Terminology Criteria for Adverse Events, with thoracic grade 3 or higher at 1 year as the primary study endpoint [10]. Thoracic grade 3 AEs were reported in 17% and 15% of patients treated with one or three fractions, respectively [10]. Thoracic grade 1–2 AEs were seen in 22% and 20% in patients with one and three fractions, respectively [10]. There were no grade 4 or 5 AEs [10]. There were no reported differences in overall quality of life measures or in pulmonary function testing between treatment arms; however, single-fraction patients had significantly better social functioning, fewer constitutional symptoms, and less dyspnea [10]. 

SAFRON II found no significant differences in adverse events [16]. There were two grade 3 or higher AEs at 1 year in the single-fraction arm and one (grade 5) in the multi-fraction arm [16]. Chest wall pain (for any grade) was not significantly different but nominally less in the single-fraction arm [16]. There were no significant differences observed in patient-reported outcomes [16].

Le et al. conducted a single-fraction SBRT study in a previously treated population [25]. There were three post-treatment deaths; all patients had received prior chemotherapy and two had received prior radiation therapy [25]. The two factors most associated with significant treatment-related toxicity were prior thoracic radiation therapy or chemotherapy, either before or after SBRT [25]. Most patients with grade 2 or greater toxicity had centrally located tumors and/or PTV > 50 cc [25]. This study suggested that 25 Gy in a single fraction was well-tolerated in previously untreated patients; however, single-fraction SBRT was too toxic in patients with prior thoracic radiation and/or chemotherapy, especially those with large or central tumors [25]. 

## 4. Discussion

This systematic review supports the use of single-fraction SBRT as a definitive treatment for early stage peripheral NSCLC or lung oligometastases with local control rates >90%, mild toxicity profiles, and favorable survival outcomes compared to multi-fraction approaches. We shared this analysis with the NCCN and are pleased to note that the updated guidelines [15] removed both tumor size and a distance of less than 2 cm from the chest wall as a factor in determining eligibility for single-fraction SBRT.

Evidence for single-fraction SBRT in these patients comes from RTOG 0915 and Roswell Park phase II randomized studies that compared single-fraction and hypo-fractionated SBRT regimens [10,11]. Both studies reported comparable local control rates, PFS, OS, late toxicities, and overall quality of life measures between the single- and multi-fraction treatment arms [10,11]. These findings align with the European Society for Radiotherapy and Oncology-American Society for Radiation Oncology (ESTRO/ASTRO) guidelines, encouraging the consideration of single-fraction SBRT during the ongoing COVID-19 pandemic [27,28].

Data on the use of single fraction since the beginning of the pandemic are limited. Prior to the pandemic, utilization of single fraction was extremely limited [19,29]. Hesitation to adopt single-fraction SBRT may have several causes: lack of familiarity with the evidence and lack of clarity about the expected toxicity [4]. Additionally, in countries with a fee-for-service payment model such as the United States, another factor may be diminished reimbursement for single-fraction regimens.

A survey of radiation oncologists in 2013 revealed that only 1% used single-fraction SBRT [29]. Consequently, the number of radiation oncologists with extensive personal experience with single fraction is limited. Anecdotally, based on number of queries that we received, more institutions have started to treat select patients with single-fraction SBRT.

Recent guidance on United Kingdom practice captures the concerns about toxicity and a lack of phase III data to justify single-fraction SBRT. The United Kingdom authors advised following the NCCN guidelines (prior to the 2022 update) and utilizing single fraction in patients with peripheral tumors that are ≤2 cm and >1 cm from the chest wall [27]. The authors cited the evidence and noted in limitations that the data are only based on phase II data. However, for tumors within 2.5 cm of the chest wall, the authors suggested three fractions while stating that the “effect of fractionation schedules on chest wall toxicity has not been investigated in prospective trials”. The prospective SAFRON II trial found that chest wall pain (for any grade) was not significantly different but nominally less in the single-fraction arm [16]. In both RTOG 0915 and the prospective trials by Singh et al., chest wall toxicity (CWT) did not exceed grade 2 in either treatment arm [10,11].

Though usually accepted to be less systematic and rigorous in evaluation of toxicity than prospective trials, multiple retrospective reviews of CWT have been performed. Bongers et al. reported on patients treated with three, five or eight fractions. CWT developed in 11.4% of patients and was severe (grade 3) in 2.0% [30]. Similarly, treating 30 or 34 Gy in a single fraction, Manyam et al. reported a comparable overall grade ≥3 CWT rate of 1.4% [31]. Both Bongers et al. and Manyam et al. concurred that CWT was associated with larger volumes of chest wall receiving doses of 30 Gy or greater.

When considering only the lesions abutting the chest wall, the 30.6% CWT rate [31] reported by Manyam et al. for single fraction is consistent with the expected grade 1–2 CWT rate of 21–34% [32,33] and rib fracture rate of up to 37% [32,34,35,36,37] observed in multi-fraction SBRT studies. For context, chronic post-operative pain from video-assisted thoracic surgery ranges from 25% to 47% [38,39]. Given that most reactions are low-grade and self-limited, location relative to the chest wall is not a contraindication for either single- or multi-fraction SBRT [31]. Consequently, the senior authors of Manyam et al. (KS and GV) do not use chest wall proximity to restrict patient eligibility for single-fraction SBRT [40]. As noted above, the updated 2022 NCCN guidelines [15] removed distance from chest wall as a factor in determining eligibility for single-fraction SBRT.

Although rare, there were three treatment-related deaths following single-fraction SBRT reported by Le et al.; all three had received prior chemotherapy and two had received prior radiation therapy [25]. Le et al. demonstrated that caution is needed when using single-fraction SBRT in patients with prior thoracic radiation and/or chemotherapy, especially with large central tumors [25]. Given the paucity of prospective studies and limited retrospective data [41], our review does not address the safety and efficacy of single-fraction SBRT for central lung tumors.

When treating pulmonary oligometastases, a review suggested favorable local control with SBRT [42]. Filippi et al. reported excellent local control and survival outcomes, making single-fraction SBRT an attractive treatment option for patients with peripherally located oligometastatic pulmonary lesions [21]. In addition, SBRT resulted in improved and durable survival benefits in the SABR-COMET trial that included 40–50% of lesions located in lungs [43,44]. However, single-fraction SBRT was not allowed in both SABR-COMET and its subsequent trial SABR-COMET-3 [43,45], while the ongoing SABR-COMET-10 trial allows 16–24 Gy of single-fraction SBRT at the treating physicians’ discretion [46]. The recently completed randomized phase II SAFRON II trial [16] showed comparable toxicity profiles, local control, and patient-reported outcomes between both single 28 Gy and four fractions of 12 Gy SBRT.

Single-fraction SBRT is an excellent option for medically inoperable patients in early stage, peripheral NSCLC patients, and those with lung oligometastases. Beyond patient convenience (one visit for treatment versus multiple), single-fraction SBRT has additional resource utilization benefits. In the context of COVID-19, the transition to single-fraction SBRT reduces risk by minimizing patient visits and reduces personal protective equipment needs. 

Unsurprisingly, in the United States, single-fraction SBRT costs less than multi-fraction SBRT. Medicare reimbursement for single-fraction SBRT is roughly half that of five-fraction SBRT. For example, in 2020, Medicare reimbursement in the western New York region for professional and technical fees was approximately USD 5600 for single-fraction SBRT, USD 7800 for three-fraction SBRT, and USD 10,000 for five-fraction SBRT. Enhanced reimbursement for multi-fraction SBRT may limit enthusiasm to adopt single fraction in the United States and other fee-for-service systems.

Furthermore, despite favorable primary tumor control seen in RTOG 0915, five-year OS remains poor at 30–40% in part due to distant metastases seen in over 40% of patients with early stage NSCLC undergoing single- or multi-fraction SBRT [11]. The optimal adjuvant therapy regimens to improve such outcomes remain unclear at this time, with numerous ongoing clinical trials evaluating the role of immunotherapy (ClinicalTrials.gov identifier: NCT03383302, NCT03446547, and NCT03110978) including PACIFIC-4/RTOG 3515 trial (NCT03833154). These studies may further provide prospective evidence on the role of single- versus multi-fraction SBRT in the setting of immunotherapy.

There are several limitations in our review. First, optimal technical approaches such as motion management and planning techniques remain unclear. For instance, RTOG 0915 allowed multiple motion managements such as abdominal compression, active breath-holding techniques, and accelerator beam gating with the respiratory cycle [11]. RTOG 0915 also allowed both 3D conformal radiation therapy (3DCRT) and intensity-modulated radiation therapy (IMRT) [11]. In SAFRON II, over 60% of patients underwent 3DCRT, and select patients utilized multiple motion management techniques [16]. Given the paucity of prospective literature comparing heterogeneous motion management and planning approaches for single-fraction SBRT, we cannot draw definitive conclusions on the role of such techniques for tumor control and toxicity outcomes. Second, although normal tissue constraints for single-fraction SBRT were previously established based on RTOG 0915 [11], more prospective data with well-defined reporting standards for toxicity outcomes are necessary for further optimization as recommended by the Hypofractionated Treatment Effects in the Clinic (HyTEC) [47,48]. Third, although the definition of central lung location has evolved over the years with the introduction of ultra-central lung location as detailed by an ongoing multi-center phase I trial [49], the definition of peripheral lung location has not been prospectively subdivided by the lesions located near versus far away from the chest wall.

The initial Indiana University and RTOG 0236 trials [50,51] did not subclassify peripheral tumors and did not report separate outcomes for lesions abutting the chest wall or at high risk for CWT. Despite this lack of prospective multi-center, long-term outcomes for these lesions at risk for CWT, both single- and multi-fraction SBRT have been utilized with a grade ≥3 CWT rate of 1–2% [30,31]. Such low rates make an adequately powered prospective trial impractical. In the absence of prospective trial data, informed decision making with patients is mandatory prior to utilizing single- or multi-fraction SBRT for lesions at risk for CWT.

## 5. Conclusions

Single-fraction SBRT for early stage peripheral NSCLC and oligometastatic lung cancer is an efficacious and well-tolerated treatment including local control rates of over 90%, favorable survival measures, and mild toxicity profiles. Given a lack of pre-specified distinction between lesions at risk for CWT versus other peripheral lesions in trials comparing single- and multi-fraction SBRT, informed decision making with patients regarding the risk of CWT is needed. This review does not support single-fraction SBRT for central lung tumors. Reducing radiation fractions while preserving treatment efficacy should be prioritized amidst the ongoing COVID-19 pandemic to mitigate disease transmission among patients and health care workers. Moreover, the encouraging outcomes, reduced resource utilization, and enhanced patient convenience of single-fraction SBRT will remain even after the current pandemic.

## Figures and Tables

**Table 1:** Study characteristics, local control, and survival outcomes for early stage non-small-cell lung cancer (NSCLC).

Study	No.	F/u (Median)	Age (Median)	Location	Stage	Dose/Fx	LC	RC	PFS	DFS	OS	Distant Failure
Hof et al., 2003 [23]	10	14.9	71	NA	T1-2N0M0	19–26 Gy/1 fx	80% at follow-up, 50% remained locally controlled	NA	LRFS 89% at 12 months, 71.1% at 24 months	NA	80% after 12 months, 64% at 24 months	Systemic metastases in 5 patients; time until diagnosis was median of 7.2 months.
Fritz et al., 2006 [22]	33	18	72	P	Stage 1	30 Gy/1 fx	94%; probability at 4 years, 83%	NA	NA	NA	Median 20.4 months. 83% at 1 year, 63% at 2 years, 53% at 3 years, 39% at 4 years.	NA
Le et al., 2006 [25]	20	18	73	C and P	T1-2N0M0	15–30 Gy/1 fx	Overall 1-year FFLR, 67%. 1-year FFLR 100% T1 tumors, 83% T2 tumors >20 Gy, 51% T2 tumors <20 Gy; 1-year FFLR all NSCLC tumors 91% >20 Gy and 54% <20 Gy	NA	NA	NA	85% at 1 year	NA
Videtic et al., 2018 (RTOG 0915) [11]	84	4 years	75	P	T1-2N0M0	34 Gy/1 fx or 48 Gy/4 fx	97% arm 1, 93% arm 2 at 1 year; 2-year primary failure rate, 2.6% arm 1, 2.2% arm 2. 5-year primary failure rate, 11% arm 1, 7% arm 2	NA	Median 2.6 years arm 1, 2.8 years arm 2. 19% arm 1, 33% arm 2 at 5 years.	77% arm 1, 84% arm 2 at 1 year. 56% arm 1, 71% in arm 2 at 2 years	85% arm 1, 91% arm 2 at 1 year. 61% arm 1, 78% in arm 2 at 2 years. Median 4.1 years arm 1, 4.6 years arm 2. 30% arm 1, 41% arm 2 at 5 years	38% arm 1, 41% arm 2.
Singh et al., 2019 [10]	98	54	71	P	T1-2N0M0	30 Gy/1 fx or 60 Gy/3 fx	95% arm 1, 97% in arm 2 at 2 years	2-year regional nodal failure rate, 8% arm 1, 16% arm 2	65% arm 1, 50% arm 2 at 2 years	NA	73% arm 1, 62% arm 2 at 2 years	13% arm 1, 19% arm 2 at 2 years

No.: number of patients; f/u: follow up; fx: fraction; LC: local control; RC: regional control; PFS: progression-free survival; DFS: disease-free survival; OS: overall survival; NA: not available; LRFS: local-recurrence-free survival; C: central; P: peripheral; FFLR: freedom from local recurrence; NSCLC: non-small-cell lung cancer; RTOG: Radiation Therapy Oncology Group.

**Table 2:** Study characteristics, local control, and survival outcomes for pulmonary metastases.

Study	No.	F/u (Median)	Age (Median)	Location	Dose/Fx	LC	RC	PFS	DFS	OS	Distant Failure
Fritz et al., 2006 [22]	25	22	65	P	30 Gy/1 fx	87%; probability at 5 years, 80%	NA	NA	NA	Median 26 months. 97% at 1 year, 73% at 2 years, 42% at 3 years, 42% at 2 years, 42% at 5 years	NA
Le et al., 2006 [25]	12	18	73	C and P	15–30 Gy/1 fx	Overall 1-year FFLR, 25%. 1-year FFLR 44% > 20 Gy	NA	NA	NA	56% at 1 year	NA
Hof et al., 2007 [24]	61	14	NA	NA	12–30 Gy/1 fx	LPFR 89% at 1 year, 74% at 2 years, 63% at 3 years	NA	NA	NA	78.4% at 1 year, 65.1% at 2 years, 47.8% at 3 years	NA
Filippi et al., 2014 [21]	67	24	NA	P	26 Gy/1 fx	93% at 1 year, 88% at 2 years; local failures, 11% of patients.	NA	72% at 1 year, 55% at 2 years	NA	85% at 1 year, 71% at 2 years. CSS 90% at 1 year, 76% at 2 years	55% of patients, at median of 8 months post-radiation
Siva et al., 2021 [16]	87	36.5	66.6 (mean)	P	28 Gy/1 fx vs 48 Gy/4 fx	1-year FFLR: 93% vs 95% at 1 year, 64% vs 80% at 3 years	NA	NA	Median 14.3 months vs. 13.2 months	95% vs 93% at 1 year, 81% vs. 67% at 3 years	Median time to distant failure: 16.0 months vs 14.5 months

No.: number of patients; f/u: follow up; fx: fraction; LC: local control; RC: regional control; PFS: progression-free survival; DFS: disease-free survival; OS: overall survival; NA: not available; C: central; P: peripheral; FFLR: freedom from local recurrence; LPFR: local progression-free rate; CSS: cancer-specific survival; RTOG: Radiation Therapy Oncology Group.

**Table 3:** Toxicity results for early stage non-small-cell lung cancer (NSCLC) and pulmonary oligometastases.

Study	Grade 1–2 Toxicity	Grade ≥3 Toxicity	Toxicity Notes
Whyte et al., 2003 [26]	NA	0%	1 COPD exacerbation, 4 pneumothoraxes s/p fiducial placement
Hof et al., 2003 [23]	NA; normal-perifocal tissue reaction, 70%	Grade >2, 0%	Some dyspnea reported, no PFT performed
Fritz et al., 2006 [22]	Grade 1 radiation dermatitis, 7%; asymptomatic radiation pneumonitis via CT at 6 months, 73%	0%	NSCLC patients pneumonitic alterations with asymptomatic, temporary pleural effusions, 24%; no changes in respiratory function
Le et al., 2006 [25]	Grade 2 pleural effusions, pneumonitis, radiation-induced atrial fibrillation, 12.5%	Grade 3 pneumonitis, 3%; Grade 4, 0%; Grade 5 pneumonitis, pleural effusions, tracheoesophageal fistula, 9%	3 post-treatment deaths; all received prior chemotherapy, 2 prior radiation therapy
Videtic et al., 2019 (RTOG 0915) [11]	1 additional Grade 1 AE arm 1, 1 Grade 2 AE (previous Grade 1) arm 2	Current rates: Grade ≥3 2.6% arm 1, 11.1% arm 2	Reported toxicities: DLCO changes, pneumonitis, PFT changes; 1 treatment-related deaths (arm 2)
Singh et al., 2019 [10]	Grade 1–2, 22% arm 1, 20% arm 2	Grade 3, 17% arm 1, 15% arm 2; no Grade 4 or 5	Better social functioning, fewer constitutional symptoms, less dyspnea arm 1.
Hof et al., 2007 [24]	Grade 1–2, 0%; normal perifocal tissue changes, 70%	Grade 3 pneumonitis requiring treatment and supplemental oxygen, 5%. Grade 4 or higher, 0%	None
Filippi et al., 2014 [21]	Grade 1, 10%.	Grade 2–3 late, 12%	Peripheral lesions with late chest wall toxicity, 9% (2 rib fractures, 4 chronic chest pain)
Siva et al., 2021 [16]	NA	3–5%; 1 patient with interstitial lung disease received multi-fraction SBRT and had grade 5 treatment-related hypoxia and radiation pneumonitis	Radiation dermatitis and esophagitis were more common in multi-fraction SBRT arm

COPD: chronic obstructive pulmonary disease; s/p: status post; NA: not available; PFT: pulmonary function test; NSCLC: non-small-cell lung cancer; CT: computed tomography scan; RTOG: Radiation Therapy Oncology Group; DLCO: diffusing capacity for carbon monoxide; AE: adverse event.
